# A Multi-Site Feasibility Assessment of Implementing a Best-Practices Meet-And-Greet Intervention in Animal Shelters in the United States

**DOI:** 10.3390/ani10010104

**Published:** 2020-01-08

**Authors:** Alexandra Protopopova, Kelsea M. Brown, Nathaniel J. Hall

**Affiliations:** 1 Animal Welfare Program, Faculty of Land and Food Systems, The University of British Columbia, Vancouver, BC V6T 1Z4, Canada; 2 National Canine Research Council, Amenia, NY 12501, USA; kbrown@ncrcouncil.com; 3 Department of Animal and Food Sciences, Texas Tech University; Lubbock, TX 79409, USA; Nathaniel.j.hall@ttu.edu

**Keywords:** adoption, animal shelter, behavior, dog, feasibility, focus group, intervention, treatment integrity, meet-and-greet, multiple baseline design

## Abstract

**Simple Summary:**

Researchers create and validate interventions to improve life-saving measures in animal shelters; however, if the animal shelters are not capable of integrating these new interventions into their existing procedures, then the benefit of research is lost. Therefore, research is also needed to assess not only the efficacy of the intervention, but also the feasibility of the new intervention for shelters. This study enrolled nine animal shelters in the United States and, using an educational session consisting of a lecture, demonstration, and role-play, encouraged them to update their current meet-and-greet procedure to the established best practice. Results showed that a single educational session was insufficient. The identified challenges included not remembering the procedures, opposing opinions of volunteers and staff, lack of resources, and a procedural drift effect in which the protocol was changed across time. These findings highlight the need for researchers to find useful ways to educate animal shelters, so that new research can be incorporated into shelter protocols and result in maximum life-saving measures of homeless companion animals.

**Abstract:**

Animal shelters must incorporate empirically validated programs to increase life-saving measures; however, altering existing protocols is often a challenge. The current study assessed the feasibility of nine animal shelters within the United States to replicate a validated procedure for introducing an adoptable dog with a potential adopter (i.e., “meet-and-greet”) following an educational session. Each of the shelters were first entered into the “baseline” condition, where introduction between adoptable dogs and potential adopters were as usual. After a varying number of months, each shelter entered into the “experimental” phase, where staff and volunteers were taught best practices for a meet-and-greet using lecture, demonstration, and role-play. Data on the likelihood of adoption following a meet-and-greet were collected with automated equipment installed in meet-and-greet areas. Data on feasibility and treatment integrity were collected with questionnaires administered to volunteers and staff followed by a focus group. We found that a single educational session was insufficient to alter the meet-and-greet protocol; challenges included not remembering the procedure, opposing opinions of volunteers and staff, lack of resources, and a procedural drift effect in which the protocol was significantly altered across time. In turn, no animal shelters increased their dog adoptions in the “experimental” phase. New research is needed to develop effective educational programs to encourage animal shelters to incorporate empirical findings into their protocols.

## 1. Introduction

Animal shelter administrators understand the need for research to improve sheltering practices as evident by the multitude of conferences that exist just within the United States that invite scientists to share their findings with staff and volunteers. Furthermore, the use of blogs, webinars, and online resources (e.g., [1]) seem to be well utilized by professionals in a wide variety of animal shelter contexts, from disease management (e.g., [2]) to community outreach (e.g., [3]). Scientists, themselves, are also increasingly concerned about effective distribution of their findings to society, industry, and clinical applications. McCormack and colleagues [4] have outlined several categories of research translation to the public: communication, dissemination, and implementation. Communication efforts deal with strategies to influence individual decision making and provide knowledge of new research in an accessible way to the broader community. Dissemination deals with a targeted distribution of information in a specific field. Finally, implementation is the use of various strategies to encourage the adoption of evidence-based programs in specific settings [4]. Recent discussions have included the duty of scientists to share data [5,6] and use their expertise to influence public policy [7,8]. Specifically, a significant challenge for scientists is to disseminate their research to practitioners, who can benefit the most [9]. Ensuring dissemination and implementation of new interventions is crucial in the medical field [10] where scientists have argued that medical advances are now less important than improving fidelity of treatment [11]. 

Similarly, animal professionals are called to influence public perception and public policy as it relates to animal welfare [12] with animal shelter-focused organizations creating numerous guidelines, position statements, and tutorials to encourage communication and dissemination [1,13,14,15]. In addition, several groups such as Maddie’s Fund, American Pets Alive! University of California Davis Koret Shelter Medicine Program, Animal Farm Foundation, and Best Friends are leading various implementation efforts in North American animal shelters to encourage overall best practices through consultation, differential funding, and activism. However, research on implementation itself within the context of animal sheltering remains sparse.

Given the focus of translational research, with an emphasis on implementation, we aimed to assess the ability of shelters to integrate a newly validated in-shelter procedure to increase dog adoptions. The selected intervention is described in detail below. We aimed to (1) assess the feasibility of animal shelters to carry out a novel best-practices procedure, and (2) identify the specific challenges which contribute to poor treatment integrity. 

### The Novel Best-Practices Procedure: Meet-And-Greet

When potential adopters are interested in adopting a dog, most shelters will allow the adopter to spend some time with their chosen dog outside of the kennel. This first-time interaction between the potential adopter and dog is commonly referred to as the “meet-and-greet.” For the current study, we selected a structured meet-and-greet as the best-practices intervention to evaluate implementation due to an already empirically established effect of this procedure in increasing adoption rates of dogs. Protopopova and Wynne [16] observed 250 meet-and-greets between shelter dogs and potential adopters at an animal shelter, and found that behavior, rather than morphology of the dogs, impacted the likelihood of subsequent adoption. The researchers found that dogs who laid down in proximity to an adopter improved their chance of adoption approximately 15-fold, but dogs who ignored the adopter’s play solicitations reduced their chance of adoption by more than 100-fold [16]. 

Following these observational findings, Protopopova and colleagues [17] experimentally assessed whether increasing appropriate and decreasing inappropriate behaviors during meet-and-greets with potential adopters influenced adoption outcomes. In Experiment 1, Protopopova and colleagues validated a brief play preference assessment to identify shelter dogs’ individual preferences for toys. The data showed that play with specific toys in the assessment predicted play in more naturalistic settings. Subsequently, this assessment was included in the experimental intervention. In Experiment 2, the dogs were randomly assigned to the experimental and control conditions and 160 interactions between these dogs and potential adopters were evaluated. Dogs in the experimental group were given a play preference assessment prior to interacting with a potential adopter, and then, when an adopter expressed interest in the dog, the nature of their interaction was structured towards the dog’s preferred mode of play. Dogs in the experimental condition engaged in less undesirable behavior, more desirable behavior, and, most important, had a 68% higher adoption rate than dogs in the control condition. Results of a questionnaire revealed that adopters did not find the structured interaction intrusive [17]. Because this meet-and-greet program was empirically-based and validated to improve adoption outcomes in dogs, it was used in the current study to assess the ability of animal shelters to alter their current protocols to established best practices following an educational session.

The aim of the study was to assess the feasibility of animal shelters in the United States to alter their current meet-and-greet procedures to match best practices. We hypothesized that an educational session consisting of a lecture, demonstration, and role-play would be sufficient in altering the current protocols. We also predicted that volunteers and staff would be able to describe the new protocol procedures accurately following implementation.

## 2. Materials and Methods

### 2.1. Overall Design

In order to retain experimental control in the applied setting of an animal shelter, a multiple baseline across shelter sites experimental design was used. A multiple baseline design involves randomly assigning various lengths of a baseline condition to each participant prior to implementing treatment [18,19]. This design is superior to a pre-post assessment as it controls for history and maturation effects [18]. Furthermore, it is superior to a traditional group design as it allows for the experimental evaluation of the intervention on a single shelter-level and does not suffer from the ethical concern of permanently keeping individuals in no-treatment conditions [20]. This design has been previously implemented in a shelter setting (across dogs [21]; across sides of a shelter [22]). By implementing this design across animal shelters, we aimed to experimentally determine whether the animal shelters could implement the best-practices procedure. Treatment integrity and feasibility of the intervention was collected through quantitative and qualitative data gathered during questionnaires and focus groups of each shelter at the completion of the study.

Each shelter experienced a baseline condition, in which they continued their standard meet-and-greet procedure, followed by an experimental condition. Each shelter was semi-randomly assigned to 2, 3, 4, or 5 months of the baseline condition followed by 3, 4, 5, or 6 months of the experimental condition. Limitations included shelter availability and travel constraints resulting in some decisions to move from the baseline to the experimental condition as well as vary the endpoint of the study. Shelter I experienced a change in administration in the first few months of the study, which resulted in the shelter withdrawing from the research. Similarly, Shelter H was suddenly and severely taxed for resources following a natural disaster, leading the shelter to withdraw from the research at a later time. Table 1 lists the months of each shelter within each condition.

### 2.2. Recruitment and Participants

Recruitment was conducted through word of mouth, utilizing established connections. As a result, a convenience sample of nine shelters was included in the study. Table 2 lists the characteristics of the shelters that were included.

During enrollment into the study, the first author visited each shelter site, explained the purpose of the study using a PowerPoint presentation (available upon request from the first author), and provided an automated data collection device (detailed description in Supplementary Materials) to be used to collect data on the proportion of adoptions following a meet-and-greet. Four shelters (Shelter A, Shelter G, Shelter H, Shelter I) chose to include a large laminated poster asking adopters themselves to operate the remote data collection device (explained below in section ‘measures’, Figure S1) and all shelters were given a small laminated poster with an explanation of the study goals. Two shelters also received the same posters translated to Spanish.

### 2.3. Intervention

When the time came for each shelter to switch to the experimental condition, the first author returned to the shelter and conducted an in-person workshop at each location to which all staff and volunteers who conduct meet-and-greets were invited. Staff were instructed to teach all other non-attending members the new procedures. The workshop included approximately 1 h of a PowerPoint presentation, approximately 1 h of role-play in the shelter’s actual meet-and-greet area with live dogs, followed by approximately 1 h of discussion of how this procedure will be best incorporated into the shelter. In addition, 100–300 toys of each type were provided: plush, rubber squeaky, rope, and tennis ball. Approximately 150–300 packets of dog treats were also provided to reduce financial burden of the intervention.

The PowerPoint presentation described the research behind the development of the meet-and-greet procedure, which included 44 slides, and three videos showing the toy preference assessment, the experimental meet-and-greet procedure, and the control procedure (to allow the viewers to compare the adopter’s behavior towards the dogs in both conditions). The presentation is available on request from the author. The role-play included visiting the actual meet-and-greet areas in the shelter. During the role-play, the experimenter asked someone to play an “adopter” and someone to play the “volunteer/staff.” First, the experimenter asked the “volunteer/staff” to let the dog off-leash in the large area full of toys, while the “adopter” was sitting on a bench. The “adopter” was asked to describe to the others how they feel about the dog’s connection to them (e.g., do they feel that the dog is paying attention to them? Do they feel a connection with the dog?). Following approximately 5 min of this interaction, the experimenter asked the “volunteer/staff” to engage in the new meet-and-greet procedure, where the dog was kept close to the “adopter” using treats, preferred toys, and a leash. The “adopter” was asked to verbally describe their feelings towards the dog yet again. This process was repeated several times with new volunteers and dogs of different behavioral needs (fearful dogs, jumpy/mouthy dogs, small dogs, etc.). During the role-play, all participants would be encouraged to verbally describe what they are observing and make comments and suggestions. During the following discussion, the experimenter and the participants brainstormed ideas that would allow the intervention to fit into the existing organizational structure of the shelter and came up with a plan to execute the intervention starting immediately.

During the workshop, the main goals were to convey the research behind the intervention, explain the underlying theory of how adopters are making choices when selecting dogs, and develop a protocol for each shelter to incorporate the new meet-and-greet procedure into their operation. During the three components of the workshop (lecture, role-play, and discussion), the following ideas were reiterated:

(1) The dog needs to be in very close proximity to the adopter, if it safe to do so;
(2) Only toys/treats that the dog likes should be present during a meet-and-greet;
(3) Having the dog lie down next to the adopter is great;
(4) The dog has to be on the leash next to the adopter;
(5) The volunteer/staff member should not be engaging with the dog, unless it is only to move the dog closer to the adopter;
(6) The volunteer/staff member should not demonstrate tricks that the dog knows during the meet-and-greet as it takes away from the interaction between the dog and the adopter;
(7) Give the adopter treats, so that s/he can give these to the dog;
(8) The main goal is to create a “connection” between the dog and the adopter;
(9) Staff/volunteers should be actively controlling the meet-and-greet to prevent the dog from becoming distracted and keep the dog’s attention on the adopter;
(10) If the dog and adopter are comfortable, place small dogs and puppies onto the adopter’s lap;
(11) Any short-term discomfort that the dog may feel in being in close proximity to a stranger will be mitigated by their long-term comfort of going to an adoptive home.

Shelters were also provided with a “cheat sheet” on the new meet-and-greet procedure (Figure S2) to edit and utilize. Some shelters elected to video record the workshop, so that the video could be posted on volunteer social media groups and used in staff meetings for dissemination.

### 2.4. Measures

Collected data included: (1) the proportion of meet-and-greets captured and the proportion that ended in an adoption (automated data collection device), and (2) feasibility and treatment integrity (questionnaire and focus group data).

We developed a device to enable remote data collection from shelters. The device (“Red Box”) was mounted near the exit of the meet-and-greet areas and collected binary yes/no responses along with a time stamp of each response. This allowed for easy collection of outcomes following a meet-and-greet that did not rely on pen-and-paper methods. In addition, the device allowed potential adopters to enter their own data thus easing the burden on staff and volunteers. Without the device, we would have needed to have staff and volunteers tally each meet-and-greet and its outcome using the pen-and-paper method, which we hypothesized would result in very low response rates. All shelters were given two automated data collection devices at the first orientation meeting, with the exception of Shelter B, Shelter C, and Shelter G, which were given one due to the smaller physical sizes of the shelters. All shelters were asked to conduct data collection during both the baseline and the experimental phases.

The third author developed devices to contain three buttons: an “ON” button, a “Taking dog home!” and “Not yet.” The “ON” button would wake the device up and enable the collection of data from the other two buttons. The activation of the “Taking dog home!” or “Not yet” buttons resulted in the recording of the time of the press. The data were recorded onto an SD card within the device. The clock in the device allowed for this time-stamping. The device contained four AAA batteries, allowing it to maintain charge for up to approximately 5 months. At any point, the experimenter could open the device, extract the SD card, and download the data. Figure S3 shows the face of the device and the electrical diagram of the device.

The operational definitions of the two buttons (“Taking dog home!” and “Not yet”) were discussed (Table 3) during the orientation meeting with each shelter site. Volunteers and staff were asked to exclude the following situations from the data collection if possible: (1) foster meet-and-greets, (2) shelter and family dog compatibility assessments, (3) volunteer-dog interactions, (4) school/class trips to see dogs. Volunteers and staff were told that collecting data on repeat visitors was fine even for the same dog-adopter dyads (e.g., adopter comes back with family member).

Upon completion of the experimental condition, an online questionnaire was administered, and the first and second authors hosted a focus group at every location. An online questionnaire was sent to supervisors to send out to all staff and volunteers (Table 4) using Qualtrics software. The focus group included discussing their answers to the questionnaire in a group meeting. Questions were related to treatment integrity, perceived efficacy, and feasibility of the new meet-and-greet procedure.

The focus groups consisted of first asking everyone to sign consent forms, then asking the attendants who had not filled out the online questionnaire to fill out the same printed questionnaire. Then the first author asked each question from the questionnaire to the group and waited for responses. If the responses were not clear, the first author asked further related questions, until confident that data saturation had occurred for that question (i.e., no additional new information was given by any participants). The accompanying second author transcribed all conversations. The questions aimed to understand how and where the meet-and-greet procedure was conducted, the challenges associated with the new procedure, any alterations made to the procedure, and perception of the efficacy of the new intervention. Additional questions were asked about the data collection process.

### 2.5. Statistical Analysis

Quantitative data from the remote data collection device in the baseline and experimental phases were compared using a paired-samples one-tailed t-test in addition to visual inspection of cumulative record data (adoption across time). Quantitative survey data were analyzed using one-way ANOVAs to compare responses across three subgroups (based on treatment integrity) of participants. Qualitative data were grouped and reported by emerging themes. Qualitative focus group data were reported by emerging themes.

### 2.6. Ethical Approval

Institutional approval from the Texas Tech University IACUC (16102-11) and IRB (2016-1000) was granted for this study.

## 3. Results

We collected quantitative data on adoption outcomes in the baseline and experimental phases of each shelter using the remote data collection devices. Additionally, we collected qualitative data on feasibility and treatment integrity using surveys and focus groups.

### 3.1. Adoption Outcomes Collected by Remote Device

Due to technical failures of the remote data collection device, adoption proportion data from the Shelter E (experimental phase) are missing, resulting in 6 shelters providing a full data set. The total number of recorded button presses was 3583 across all shelters (Shelter A = 186, Shelter B = 1052, Shelter C = 570, Shelter D = 997, Shelter F = 448, and Shelter G = 330). To very roughly estimate the proportion of captured meet-and-greets by the remote device in each shelter, we divided the actual captured number of meet-and-greets by the estimated theoretical total of meet-and-greets in each shelter using the following assumptions: 60% of shelter intake are dogs and 70% of dog adoptions are preceded by a meet-and-greet. With these assumptions, we captured a median of 25% of all conducted meet-and-greets, with dramatic variance between shelters (Shelter A = 2%, Shelter B = 100%, Shelter C = 25%, Shelter D = 28%, Shelter F = 18%, and Shelter G = 25%).

The median adoption proportion in baseline phase was 0.46 (interquartile range [IQR] = 0.05) and in the experimental phase was 0.48 (IQR = 0.06). Visual analysis of raw data confirmed the lack of effect in every shelter (cumulative records of adoptions across time in each shelter site is available upon request from the authors). Given that we captured so few meet-and-greets, we elected to not analyze these data any further.

### 3.2. Feasibility and Treatment Integrity

Qualitative data on feasibility and treatment integrity were collected using questionnaires as well as focus groups at the end of the experimental phase for each shelter.

#### 3.2.1. Questionnaire Data

A total of n = 43 participants completed the questionnaire, with n = 1 who reported not conducting meet-and-greets as part of their job; therefore, the total number of responses was n = 42. The reported roles of the participants ranged from volunteer (n = 16), animal care attendant/associate (n = 6), manager (n = 3), behavior/welfare staff (n = 8), and adoption/matchmaker staff (n = 9). The participants were separated into three categories based on Q5 (Table 4). “Describe the experimental meet-and-greet procedure”): a) those that reported three or more accurate statements and zero wrong statements in describing the procedure (n = 12), b) those that reported at least one but fewer than three accurate statements combined with at least one wrong statement (n = 15), and c) those that did not report any accurate statements (n = 15). In other words, only 12 of the 42 participants could accurately describe the meet-and-greet procedure. The first subgroup was further analyzed for subsequent treatment integrity. The two inaccurate subgroups created natural control groups to better investigate feasibility and perceived efficacy questions.

Out of the 12 participants that accurately described the experimental meet-and-greet procedure (Q5), eight (67%) reported following the procedure (Q6), with the remaining participants reporting some alterations depending on specific situations. When asked to rate the likelihood that they followed the procedure (Q8), five (42%) reported that they were very likely, four (33%) reported that they were somewhat likely, and three (25%) reported that they were unlikely to follow the procedure. Three (25%) reported that they carried out the procedure every time, eight (67%) reported carrying out the procedure most of the time, and one (8%) reported carrying out the procedure some of the time (Q10).

When asked what they liked most about the program (Q13), the 12 participants in the accurate group reported that they believed that the adopters had a better experience (n = 4), enjoyed having a structure to the meet-and-greet (n = 3), used the time for additional training or behavioral evaluation of the dogs (n = 4), and enjoyed educating adopters (n = 1). When asked what they liked least about the program (Q14), the 12 participants reported difficulties in carrying out the procedure when multiple adopters are present (n = 1), time constraints (n = 2), remembering the procedure (n = 2), feasibility (n = 3), feeling like they were forcing the dog into an interaction (n = 1), and a lack of compliance from others (n = 1). When asked what they would improve (Q15), the participants reported increasing flexibility in the procedure (n = 1), more information on how to adjust the procedure given specific constraints (n = 3), and more reminders about the procedure to increase compliance (n = 2).

Participants in the subgroup which reported only wrong components had a higher perceived efficacy (Q12) of the procedure compared to the other two subgroups (F = 4.1, df = 2, *p* = 0.025). The mean perceived efficacy for the accurate subgroup was 2.4 (SD = 0.8), for the partially accurate subgroup was 2.2 (SD = 1.0), and for the wrong subgroup was 3.2 (SD = 1.1). Question 4 was removed from analysis as only one participant provided an answer. Satisfaction (Q7) with the program did not differ across the three subgroups of participants (F = 0.89, df = 2, *p* > 0.05). Also, the participants in the three subgroups did not differ in how challenging (Q9; F = 0.34, df = 2, *p* > 0.05) or reasonable they found the program (Q11; F = 1.2, df = 2, *p* > 0.05).

#### 3.2.2. Focus Group Data

All shelters except Shelter A (management cancelled visit due to time constraints) participated in the focus groups. The number of participants ranged from two to 11 people (average of 5.2) and included management, staff, and volunteers. Different shelter sites varied in their emphasis of various themes; however, several consistent themes emerged:

1. Lack of knowledge about experimental procedure or the study altogether,
2. No memory of the details of the experimental procedure while remembering the purpose of the procedure,
3. Misremembering specific details of the experimental procedure and carrying out an erroneous procedure,
4. Active disobedience due to opposing views,
5. Lack of available resources and time, and
6. Satisfaction that the experimental procedure is empirically validated.

The first three themes were the most common across animal shelters. Because of high staff turn-over and perhaps lack of consistent communication, many participants reported not being aware of study procedures at all. Others remembered the purpose of the study (repeated the “ideas” as reported in the methods section) but lacked the memory of how to implement the procedure with adopters. The authors were surprised to learn that in most animal shelters where the participants reported remembering and following the experimental procedure, they reported erroneous—and sometimes even potentially harmful—details of the procedure. For example, several reported “cutting off” the interaction between an adopter and dog after 8 min in order to encourage the adopter to make a quick decision. Another participant reported that the experimental procedure included encouraging a dog to jump on adopters. Other erroneous details included who holds the dog’s leash, which dogs can participate in the study, and what constitutes an “adoption.”

Another common theme that emerged was active disobedience due to personal beliefs. Common opposing beliefs included ideas that (1) the dogs need to be shown in a natural state rather than in a structured manner, (2) it is important to demonstrate your personal relationship with the dog to show the adopter what the dog is capable of, (3) all dogs need a personalized approach and so structured programs do not work, and (4) it was dishonest to show the dog in a more positive light.

Another theme was the frustration by management that they did not have the time or resources to alert and monitor staff and volunteers in conducting the experimental procedure. Staff and volunteers reported not having enough time to remember and carry out the procedure during hectic days. Several staff indicated that consistent daily reminders of the procedure would have been beneficial.

Finally, many reported satisfaction in that the experimental procedure was empirical in nature and that they enjoyed having more research to back up shelter operations. In addition, many reported enjoying a more structured approach to something that they felt was typically unstructured and largely forgotten.

## 4. Discussion

Animal shelter administrators are increasingly looking to scientists to provide empirical programs in order to improve animal shelter practices and improve life-saving measures. Whereas researchers often focus on developing and evaluating programs within animal shelters, there is a stark lack of research on ensuring implementation of best practices. However, without implementation, development of new programs is arguably useless. Previously, scientists have found that implementation challenges rather than innovations in procedures result in the failure of novel programs [11]. In the current study, we evaluated whether a single workshop consisting of a lecture, role-play, and discussion could ensure implementation of an empirically-based best-practices procedure of the first meeting between a potential adopter and their dog of interest in animal shelters within the United States.

Questionnaire and focus group data showed very low treatment integrity across all shelters, suggesting that the educational session was not sufficient to change the existing meet-and-greet procedure of each shelter. In addition to low treatment integrity, the animal shelters also varied greatly in the numbers of captured meet-and-greets by the remote device, suggesting large variance in the ability of different shelters to implement a new procedure. Differences across shelters are substantial, with variation in day-to-day procedures [23,24] and differences in animal health [25,26] and staff wellbeing [27]. It is likely that differences in budget, location, and leadership all affect the ability and interest of animal shelters to implement novel programs.

The field of Implementation Science developed several conceptual models that can describe the factors that influence implementation efforts. One such framework is the Exploration, Preparation, Implementation, and Sustainment (EPIS) model [28,29]. Within this framework, implementation relates to the outer context (e.g., societal environment, inter-organizational factors), inner context (e.g., leadership, quality of monitoring and support, organizational characterizations, and personal attitudes), innovation or program factors (i.e., how the new best practice fits into the current procedures), and bridging factors (i.e., quality of the partnership between the research institution and the animal shelter) [29]. Thus, the barriers to implementation can be further described as relating to the distal level, organizational level, and proximal level [30].

At the distal level are sociopolitical contexts, which may include societal expectations that animal shelters need to use empirically-based programs and that increasing dog adoptions is a needed enterprise. Barriers at this level may include a lack of emphasis on university-animal shelter collaborations or a lack of overall concern with programs being empirically-evaluated. However, our results do not indicate that these kinds of barriers were significant. In fact, participants reported liking that the experimental procedure was scientifically developed and assessed. Shelter administrators and staff welcomed a collaboration with a university and looked forward to the data outcomes.

Barriers at the organizational level may include the culture of the organization, leadership, available resources, and the readiness to implement new procedures. One common theme in the responses within our study was that many staff and volunteers were not aware of the experimental study procedure, likely due to high turnover rates. Thus, many people did not participate in the training session and had to rely on management staff to teach them the new procedures, which largely did not happen. Likely, a lack of time and resources prevented management from ensuring that all new employees also follow new procedures. In support of this hypothesis, several participants who occupied management roles reported wanting continuous reminders and a way to track compliance of their staff.

Barriers at the proximal level included communication, training, and making decisions based on previous data rather than personal opinion. Our data suggests that these proximal barriers were particularly significant. A common theme, and perhaps one that was most detrimental, was misremembering the details of the new experimental procedure. As staff and volunteers were trained by either management or peers, and subsequently were asked to train other peers, the details of the procedure were either lost or significantly altered. A striking example of this effect occurred in one shelter site, where at the end of the study period staff and volunteers were asking potential adopters to stop interacting with their chosen dogs after 8 min had elapsed. The misunderstanding likely occurred after management staff mistakenly highlighted the fact that previous research has found that adopters spend 8 min, on average, interacting with their chosen dog prior to making a decision; this detail, then, made it to the guidelines resulting in all volunteers and staff erroneously truncating the meetings. This example is particularly striking as it may have actually reduced the likelihood of adoption, perhaps from annoyed adopters leaving the shelter. The problem of misremembering may not only have a detrimental effect on the implementation of best-practice procedures, it may also eradicate the trust that staff and volunteers have in animal welfare science and applied researchers. Several participants reported not feeling comfortable with the supposed procedure and feeling that it did nothing to improve adoption, only reduce adoption likelihood. Therefore, ensuring that this procedural drift effect is minimized should be at the forefront of successful implementation programs. Perhaps accuracy in the procedure may be maintained through continuous reminders of not only the purpose of the procedure but also all of the details of the procedure. Additionally, it may be useful to verify accuracy by continuously asking all staff and volunteers to report back on exactly what they are doing and adjusting these implemented procedures as they inevitably start straying from the original guidelines.

Another emerging theme was found to be active noncompliance due to opposing views as well as opposing personal experiences. For example, many volunteers felt that it was important to demonstrate the attachment between them and the dog to the potential adopter, so that the adopter could imagine that a future bond with the chosen dog is possible. However, our previous research suggests that adopters require that the dog demonstrates some affiliative behaviors towards them rather than simply seeing a dog demonstrate these affiliative behaviors towards someone else [16], although a direct experimental comparison of the two interaction types is lacking. Another common reason for noncompliance was due to opposing personal experiences. For example, several participants in the focus groups relayed personal stories of cases in which the best-practices procedure would not work. These case stories typically involved very emotional and striking situations in which someone could have been bitten or a dog could have been returned to the shelter. These opposing views may have resulted from selectively focusing on exceptions and emotionally-charged situations rather than average situations.

Our findings support previous research in identifying the barriers to program implementation and connect it to the field of animal sheltering. Following from our findings, new implementation programs need to directly address barriers at the organizational and proximal levels. The new procedures must be empirical in nature, fit into the existing organizational climate, ensure sufficient resources and staff readiness to implement the procedure, as well as ensure that the animal shelter has a leader to be the champion of the new procedure. Additionally, adequate communication, training, coaching, and assessment from the leadership team must be in place to reduce proximal barriers.

## Figures and Tables

**Table 1:** Multiple baseline design across animal shelter sites.

Shelter Site	Months								
	1	2	3	4	5	6	7	8	9
Shelter A	BL	BL	EXP	EXP	EXP	EXP	EXP		
Shelter B	BL	BL	EXP	EXP	EXP	EXP			
Shelter C	BL	BL	BL	EXP	EXP	EXP	EXP	EXP	EXP
Shelter D	BL	BL	BL	BL	EXP	EXP	EXP	EXP	
Shelter E	BL	BL	BL	BL	BL	EXP	EXP	EXP	EXP
Shelter F	BL	BL	BL	BL	BL	EXP	EXP	EXP	
Shelter G	BL	BL	BL	BL	BL	EXP	EXP	EXP	EXP
Shelter H (withdrew)									
Shelter I (withdrew)									

“BL”—baseline phase, in which shelters were asked to continue their regular meet-and-greet procedure; “EXP”—experimental phase, in which shelters were asked to carry out the new meet-and-greet procedure. The names of the animal shelters have been altered to protect their privacy.

**Table 2:** Characteristics of participating animal shelters at the time of the study.

Shelter Site	Region	Type	Approx. Yearly Intake	Approx. Live-Release Rate *	Original Meet-And-Greet Procedure		
					Leash/No Leash	Indoor/Outdoor Area	Staff/Volunteer Supervision
Shelter A	South West	Municipal	40,000	87%	No leash	Indoor and outdoor (large)	Yes
Shelter B	West Coast	Private	5000	97%	No leash	Indoor and outdoor (large)	Yes
Shelter C	New England	Private	9000	80%	No leash	Outdoor (large)	No
Shelter D	South West	Private	17,000	75%	No leash	Indoor and outdoor (large)	Yes
Shelter E (equipment malfunction)	West Coast	Municipal	40,000	80%	Leash	Outdoor (bench)	Yes
Shelter F	West Coast	Municipal	10,000	89%	No leash	Indoor	Yes
Shelter G	South Central	Municipal	9000	46%	No leash	Indoor	No
Shelter H (withdrew)	South Central	Private	12,000	97%	No leash	Outdoor (small)	Yes
Shelter I (withdrew)	South Central	Municipal	20,000	95%	No leash	Outdoor (small)	No

The names of the animal shelters have been altered to protect their privacy. * The Live-Release Rate is a standardized measure, which can be interpreted as the percentage of animals that exit the shelter alive.

**Table 3:** Operational definitions of the binary response following a meet-and-greet on the remote data collection device.

Adoption (“Taking Dog Home!”)	Non-Adoption (“Not Yet”)
Application is approved and the dog leaves the shelter	Application is submitted, but adopter does not want to make a final decision in that moment
Application is approved, dog leaves the shelter, but adopter is asked to come back to finish process (or foster-to-adopt contract)	Adopter wants to bring back a family member/friends/family dog
Application is approved, but the dog cannot leave the shelter yet	Adopter does not submit an application
Adopter had the intention of adopting, but the application was not approved	
Application is approved, but the adopter leaves without the dog to come back later the same day (to gather supplies, get a crate, etc.)	

**Table 4:** Questions asked during the focus group.

Question	Type	Theme
Q1. Please write the name of your shelter.	Open ended	Information
Q2. What is your role in the shelter?	Open ended	Information
Q3. Do you assist potential adopters with selecting or meeting their prospective dogs in the shelter?	Binary	Information
Q4. If you mentioned that you do not assist adopters. In that case, did you hear about the new meet-and-greet program running in your shelter from others? If so, what did you hear about it? Please describe the good and the bad!	Open ended	Perceived efficacy
Q5. In your own words, can you describe the new meet-and-greet procedure that you were asked to do with potential adopters?	Open ended	Treatment integrity
Q6. Did you end up conducting the procedure differently than how you were asked? If so, what were the differences? What was/were the reason(s) for these differences? (This question is not intended to be judgmental! We are interested in what worked and what did not work in your shelter. Thank you again for your honesty!)	Open ended	Treatment integrity
Q7. Overall, how satisfied or dissatisfied were you with this the new way of conducting the meet-and-greets?	1–7 scale	Feasibility
Q8. How likely were you to follow the procedures of the new meet-and-greet program with potential adopters these last few months?	1–10 scale (recoded into 3 categories)	Treatment integrity
Q9. How challenging was it to carry out the new meet-and-greet procedure?	1–5 scale	Feasibility
Q10. How often did you carry out the new meet-and-greet procedure with potential adopters?	1–5 scale	Treatment integrity
Q11. How reasonable or unreasonable was the new meet-and-greet procedure?	1–7 scale	Feasibility
Q12. To your best knowledge, how well did this new meet-and-greet procedure work to increase adoption likelihood (in your opinion)?	1–5 scale	Perceived efficacy
Q13. What did you like most about the new meet-and-greet program?	Open ended	Feasibility
Q14. What did you like least about the new meet-and-greet program?	Open ended	Feasibility
Q15. How could the meet-and-greet program be improved, in your opinion?	Open ended	Feasibility
Q16. Please let me know any other thoughts that you may have about this program.	Open ended	Feasibility

## Supplementary Materials

The following are available online at https://www.mdpi.com/2076-2615/10/1/104/s1, Figure S1: A provided poster asked shelter visitors to operate the remote data collection device following a meet-and-greet, Figure S2: A provided “cheat sheet” for the animal shelters to remember the key features of the meet-and-greet intervention, Figure S3: The face and electrical diagram of the remote data collection device.
