# Supplementary material for: A Multi-Site Feasibility Assessment of Implementing a Best-Practices Meet-And-Greet Intervention in Animal Shelters in the United States
Source: Animals (Basel). 2020 Jan 8;10(1):104. doi: 10.3390/ani10010104 (PMC7023286; doi:10.3390/ani10010104)
Supplement: Supplementary file 1 [file animals-10-00104-s001.pdf]

A Multi-Site Feasibility Assessment of Implementing a Best-Practices Meet-And-Greet Intervention in Animal Shelters in the United States

Alexandra Protopopova [1,*], Kelsea M. Brown [2] and Nathaniel J. Hall [3]

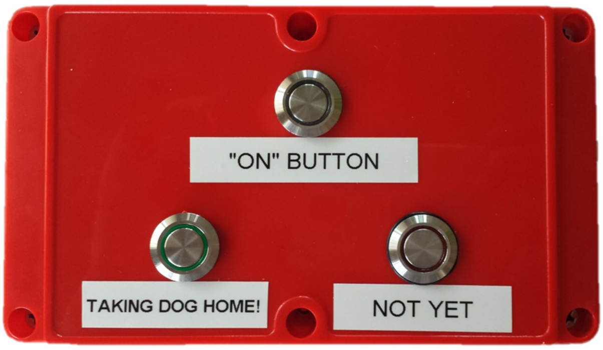

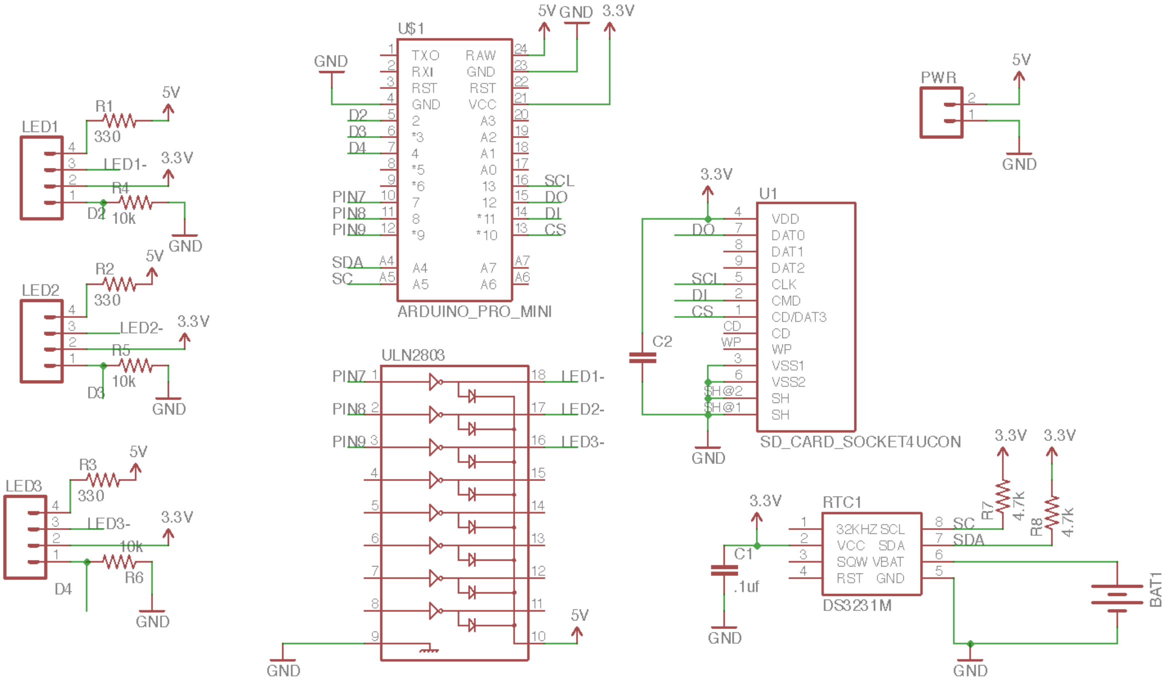

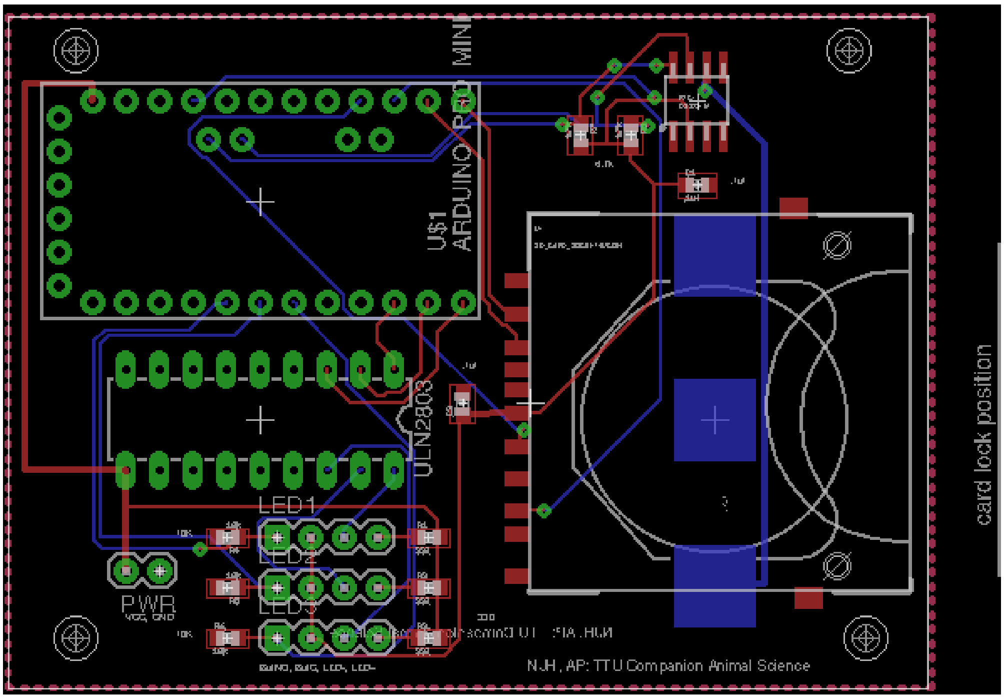

Figure S1. The face and electrical diagram of the remote data collection device.

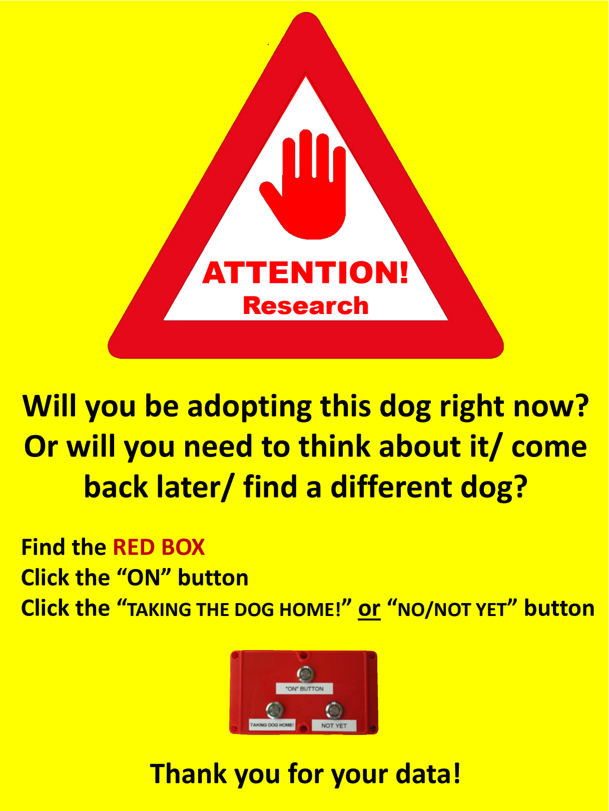

Figure S2. A provided poster asked shelter visitors to operate the remote data collection device following a meet-and-greet.

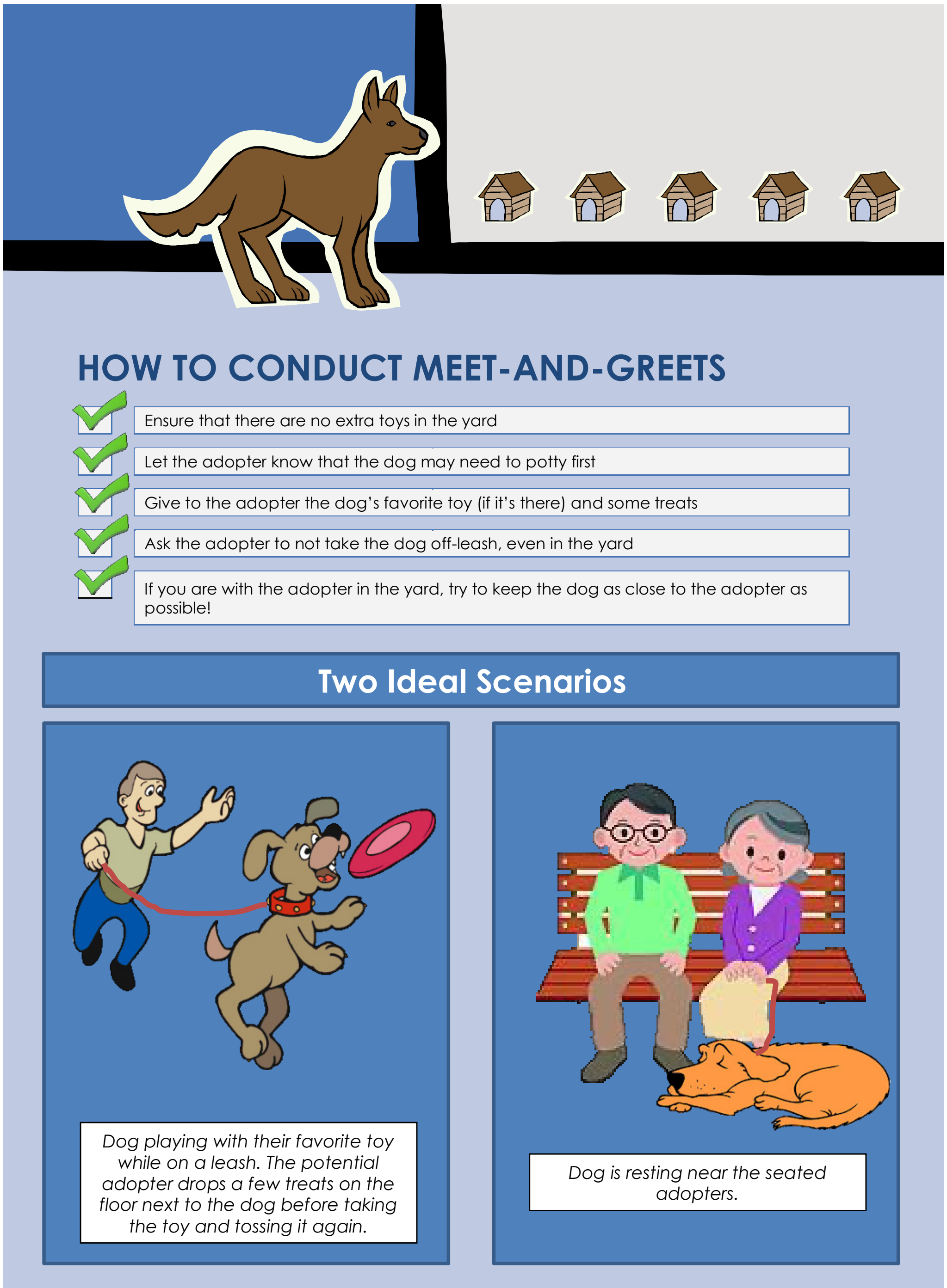

Figure S3. A provided "cheat sheet" for the animal shelters to remember the key features of the meet-and-greet intervention.
